# Meesmann Corneal Dystrophy; a Clinico-Pathologic, Ultrastructural and Confocal Scan Report

**Published:** 2010-04

**Authors:** Mohammad-Ali Javadi, Mozhgan Rezaei-Kanavi, Atefeh Javadi, Nima Naghshgar

**Affiliations:** Ophthalmic Research Center, Shahid Beheshti University of Medical Sciences, Tehran, Iran

**Keywords:** Meesmann, Confocal, Electron Microscopy

## Abstract

**Purpose:**

To report the microstructural features of Meesmann corneal dystrophy (MCD) in two patients.

**Case Report:**

The first patient was a 10-year-old boy who presented with bilateral visual loss, diffuse corneal epithelial microcystic changes, high myopia and amblyopia. With a clinical impression of MCD, automated lamellar therapeutic keratoplasty was performed in his left eye. Histopathologic examination of the corneal button disclosed epithelial cell swelling and cyst-like intracytoplasmic inclusions. The cells contained moderate amounts of periodic acid-Schiff-positive and diastase-sensitive material (glycogen). Transmission electron microscopy revealed numerous vacuoles and moderate numbers of electron-dense membrane-bound bodies in the cytoplasm, similar to lysosomes, some engulfed by the vacuoles. The second patient was a 17-year-old female with a clinical diagnosis of MCD and episodes of recurrent corneal erosion. On confocal scan examination of both corneas, hyporeflective round-shaped areas measuring 6.8 to 41.4 μm were seen within the superficial epithelium together with irregular and ill-defined high-contrast areas in the sub-basal epithelial region. The subepithelial nervous plexus was not visible due to regional hyperreflectivity.

**Conclusion:**

This case report further adds to the microstructural features of Meesmann corneal dystrophy and suggests confocal scan as a non-invasive method for delineating the microstructural appearance of this rare dystrophy.

## INTRODUCTION

Meesmann corneal dystrophy (MCD) is a rare bilateral corneal epithelial disorder which appears in the first or second year of life and was first described by Pameijer in 1935.^1^,^2^ The pattern of inheritance is autosomal dominant but an autosomal recessive form has also been reported.^1^ On slitlamp biomicroscopy, the lesions appear as punctate, bubble-like, round to oval opacities in the corneal epithelium. On histopathology, the dystrophic epithelium is characterized by cellular swelling, cyst-like inclusions, and cytoplasmic vacuoles. The cysts appear to contain degenerated cell debris which is periodic acid-Schiff (PAS) positive.^3^ Although the cells contain PAS-positive material, this may not be excessive glycogen as was previously believed; the material has been reported to be a dense intracellular substance of unknown composition.^1^ On electron microscopic examination, an electron-dense and amorphous “peculiar substance” has been reported in the cytoplasm of epithelial cells. Deposition of the substance in the epithelium leads to cyst formation and cell death followed by rapid regrowth of the epithelium.^3^

Confocal scan is a non-invasive diagnostic tool for rapid evaluation of all corneal layers and *in vivo* diagnosis of corneal disorders.^4^,^5^ The reported confocal microscopic features of MCD^6^,^7^ include well delineated cystic lesions containing hyperreflective points,^7^ hyporeflective areas in the basal epithelial layer, large elongated intraepithelial clefts and reflective spots within the hyporeflective areas.^6^ Reports on the microstructural features of MCD are limited in the literature and there are only two reports^6^,^7^ on confocal scan findings of this rare dystrophy. We believe that this report adds to the microstructural information available for MCD.

## CASE REPORTS

### Case 1

**Figure 1:**
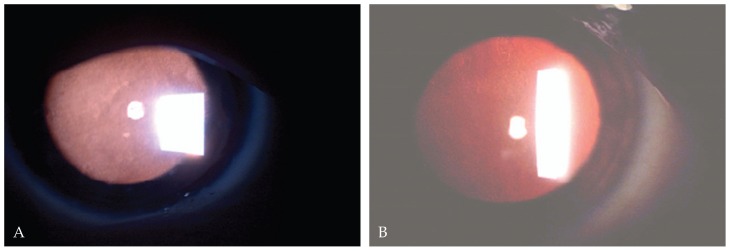
Diffuse intraepithelial microcystic lesions on slitlamp biomicroscopy visible by retroillumination in the first **(A)** and second **(B)** patient.

A 10-year-old boy presented with bilateral decreased vision since the age of three. His parents were consanguineous but normal on ophthalmic examinations. Best-corrected visual acuity (BCVA) was 20/160 and 20/800 in his right and left eyes with correction of −12.00 and −13.00 sphere, respectively. On slitlamp biomicroscopy, diffuse intra-epithelial microcystic changes were present within the entire corneal epithelium (Fig. 1A). Intraocular pressure (IOP) was normal and funduscopic examination disclosed pathologic myopic changes in both eyes. The clinical diagnosis was MCD associated with amblyopia due to high myopia. To improve visual acuity and anterior corneal clarity,^8^ the patient underwent automated lamellar therapeutic keratoplasty (ALTK) with a thickness of 250 μm in his left eye.

**Figure 2:**
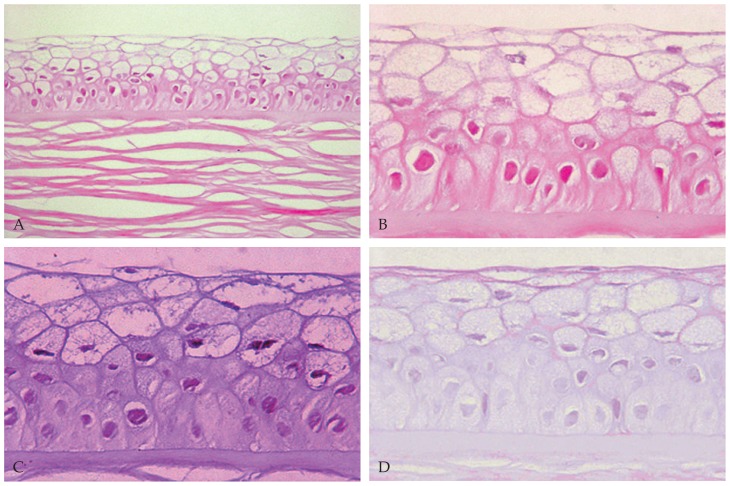
Abnormal corneal epithelium with cellular swelling and intracytoplasmic cyst-like inclusions (A & B) on Hematoxylin & Eosin staining (**A:** magnification × 400, **B:** magnification ×1000). Note the presence of moderate amounts of periodic acid-Schiff-positive **(C)** and diastase-sensitive **(D)** material within the abnormal epithelial cells (magnification ×1000).

**Figure 3:**
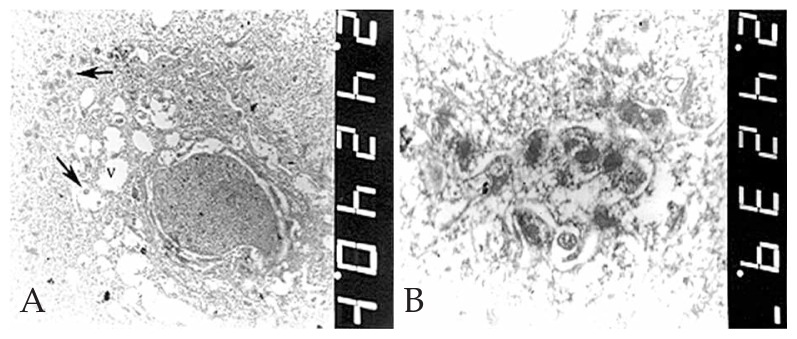
Transmission electron microscopy: **A)** Numerous vacuoles (V) of variable size and clusters of electron-dense bodies (arrows) within the cytoplasm of an epithelial cell (magnification ×4400). **B)** Electron-dense, membrane-bound bodies similar to lysosomes, some within the vacuoles in the cytoplasm of epithelial cells (magnification ×20,000).

The corneal button was sent in 10% formalin to the pathology laboratory. After bisecting the specimen, one half was processed and embedded in paraffin wax. Sections were prepared and stained with Hematoxylin and Eosin (H&E) for studying the general morphology and by PAS sequence with and without diastase to identify glycogen.^9^ The histopathological sections were examined by light microscopy (Olympus BX43, Olympus Co., Tokyo, Japan). The other half of the specimen was sent in 2.5% glutaraldehyde to the electron microscopy laboratory for transmission electron microscopy (EM 900, Zeiss, Germany). Histopathological examination disclosed a partial-thickness cornea with abnormal-appearing epithelium consisting of numerous intracytoplasmic cyst-like inclusions together with cellular swelling (Figures 2A, 2B). The cells contained moderate amounts of PAS-positive (Fig. 2C), diastase-sensitive (Fig. 2D) material consistent with glycogen. Other corneal layers were unremarkable. Transmission electron microscopic examination disclosed numerous and variable-sized vacuoles within the cytoplasm in all epithelial layers (Fig. 3A). Moderate numbers of electron-dense and membrane-bound intracytoplasmic bodies similar to lysosomes were also noted, some within the vacuoles (Fig. 3B). No abnormal findings were noted elsewhere. The histopathologic and electron microscopic findings confirmed the clinical diagnosis of MCD.

### Case 2

A 17-year-old female presented with foreign body sensation and pain in both eyes since 3 years ago without any significant medical or family history. BCVA in her right and left eyes was 20/40 and 20/50, respectively with refractive error of −0.5 sphere in both eyes. On retroillumunation by slitlamp biomicroscopy, there were diffuse (limbus to limbus) intra-epithelial microcystic lesions together with regional haze in both corneas (Fig. 1B) but other corneal layers were unremarkable. IOP and funduscopic examinations were within normal limits. The clinical features were characteristic for MCD.

After topical anesthesia, confocal scan 3.0 (Nidek Technology, Padova, Italy) was performed on both eyes using methylcellulose as a coupling agent between the front lens (40×, 0.75 objective lens) and the surface of the cornea. The automatic full thickness and epithelial modes were used to capture images from all corneal layers with particular attention to the anterior parts of the involved cornea. The manual analytic software of the confocal scan was utilized to measure the abnormal confocal findings.

**Figure 4:**
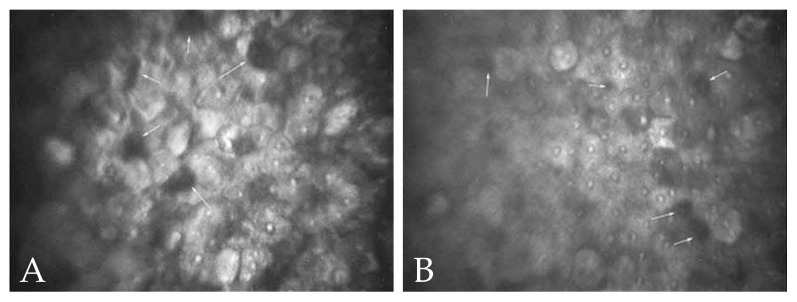
Round to oval, hyporeflective intracytoplasmic cystic structures (arrows) in the superficial corneal epithelium on confocal scan examination of the second case (A&B).

**Figure 5:**
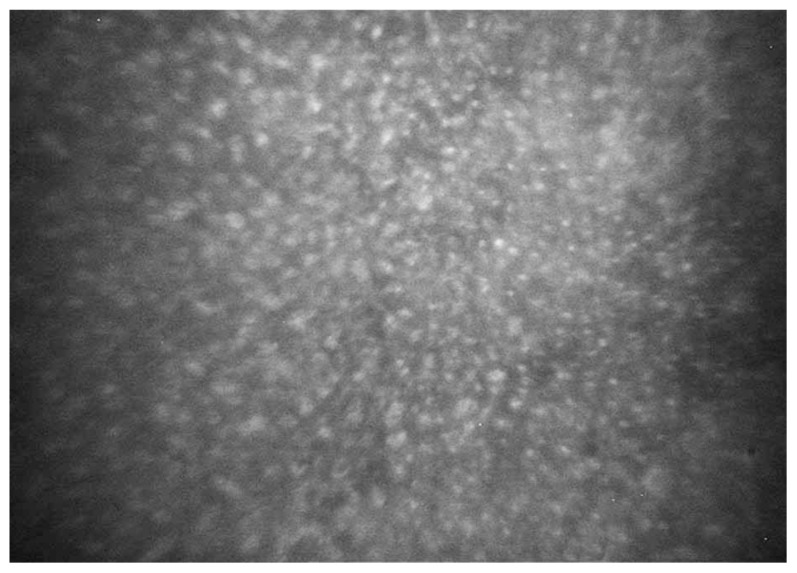
Note the presence of diffuse hyperreflective spots in the basal corneal epithelium on confocal microscopy.

**Figure 6:**
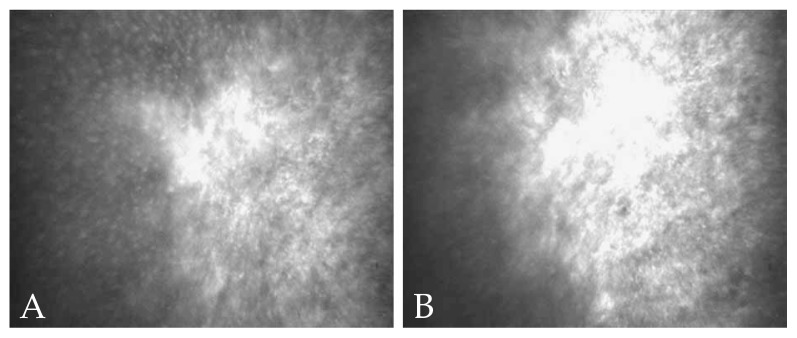
Note the presence of irregular and poorly-defined high contrast areas in the sub-basal epithelial region and foci of sub-epithelial fibrosis (A&B) on confocal microscopy.

Confocal scan examination of both corneas disclosed scattered and well-defined round to oval hyporeflective intracytoplasmic areas measuring 6.8 to 41.4 μm in their largest diameter within the superficial corneal epithelium (Figures 4A & 4B), diffuse hyperreflective spots in the basal epithelium (Fig. 5), irregular and poorly-defined high contrast areas in the sub-basal epithelial region and foci of sub-epithelial fibrosis (Fig. 6). A few hyperreflective lesions containing high contrast spots, corresponding to cell nuclei, were also present. The subepithelial nerve plexus was not visible because of the regional hyperreflectivity. No abnormal finding was noted in the rest of corneal stroma and the endothelium. The confocal microscopic features were consistent with the clinical diagnosis of MCD.

## DISCUSSION

The histopathologic features of the excised corneal button in our first case were similar to those described by Chiou et al:^10^ an increase in corneal epithelial thickness, presence of intraepithelial microcysts, and increased amounts of intracellular glycogen. The ultrastructural appearance was also similar to that reported by Nakanishi et al^11^ in terms of presence of intense intracytoplasmic vacuolation and formation of lysosome-like, electron-dense bodies within the cytoplasm of epithelial cells. These features are distinctly different from the electron-dense “peculiar substance”^1^ or the electron-dense fibrillogranular material^10^ reported earlier.

We observed hyporeflective cystic structures of various sizes in the superficial corneal epithelium on confocal scan examination of the second case, which is similar to findings previously reported for MCD.^6^,^7^ The presence of diffuse hyperreflective spots in the basal epithelium, high contrast sub-basal epithelial areas and subepithelial fibrosis were new findings in our study not previously described. It has been suggested that hyperreflective spots within cystic lesions may correspond to cell nuclei,^7^ however they may be due to accumulation of intracytoplasmic lysosomes containing degenerated cellular material. We assume that the irregular high contrast areas in the sub-basal epithelial region correspond to nonspecific irregular thickening of the epithelial basement membrane which may be seen in most cases of MCD.^3^

The diagnosis of MCD is based on clinical findings such as bilateral limbus to limbus microcystic intraepithelial changes on high power slitlamp biomicroscopy,^12^ and may be further confirmed microstructurally with light and electron microscopy^1^ or through confocal microscopy.^6^ Since surgical intervention such as lamellar or penetrating keratoplasty is not indicated in the majority of patients with MCD,^6^,^8^ *in vivo* confocal microscopy can provide a non-invasive method for confirming the diagnosis.

In conclusion, this report could further add to the microstructural information available on Meesmann corneal dystrophy and present new confocal microscopic features of this rare dystrophy.
